# (1,5-Diphenyl­thio­carbazonato-κ*S*)trimethyl­tin(IV)

**DOI:** 10.1107/S1600536812047216

**Published:** 2012-11-24

**Authors:** Karel G. Von Eschwege, Jannie C. Swarts, Manuel A. S. Aquino, T. Stanley Cameron

**Affiliations:** a Department of Chemistry, University of the Free State, PO Box 339, Bloemfontein, 9300, South Africa; b Department of Chemistry, St Francis Xavier University, PO Box 5000, Antigonish, Nova Scotia B2G 2W5, Canada; c Department of Chemistry, Dalhousie University, Halifax, Nova Scotia, B3H 4J3, Canada

## Abstract

In the title compound, [Sn(C_13_H_11_N_4_S)(CH_3_)_3_], the Sn^IV^ atom is coordinated by an S atom from the 1,5-diphenyl­thio­carbazonato (*L*) ligand [Sn—S 2.4710 (6) Å] and by three methyl groups [Sn—C 2.123 (3)–2.130 (2) Å] in a distorted tetra­hedral geometry. The aromatic rings of the *L* ligand form a dihedral angle of 2.1 (1)°.

## Related literature

For general background to dithizone and dithizonato metal complexes, see: Irving (1977 ▶). For the synthesis of dithizone, see: Pelkis *et al.* (1957 ▶). For structural aspects of dithizone and its oxidation products and observed solvatochromism and concentratochromism, see: Von Eschwege *et al.* (2011*a* ▶). For related ligand and complex structures, see: Harrowfield *et al.* (1983 ▶); Kong & Wong (1999 ▶); Herbstein & Schwotzer (1984 ▶); Fernandes *et al.* (2002 ▶); Von Eschwege *et al.* (2008 ▶); Laing *et al.* (1971 ▶). For electrochemical studies of dithizone and its Hg complex, see: Von Eschwege & Swarts (2010 ▶); Von Eschwege *et al.* (2011*b* ▶). For femto second laser spectroscopy studies on a photochromic dithizonatomercury complex, see: Schwoerer *et al.* (2011 ▶). For the weighting scheme, see: Carruthers & Watkin (1979 ▶).
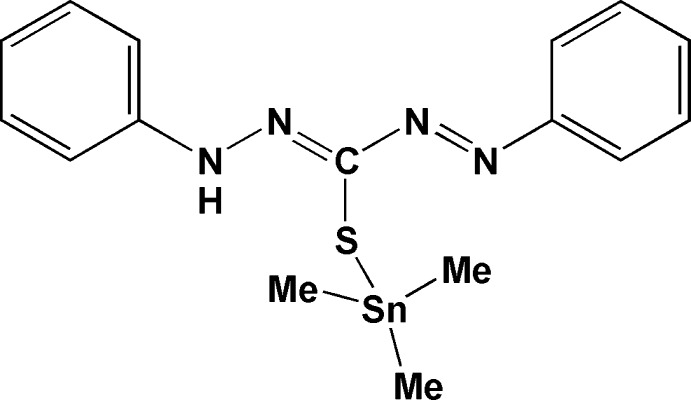


## Experimental

### 

#### Crystal data

[Sn(C_13_H_11_N_4_S)(CH_3_)_3_]
*M* *_r_* = 419.11
Monoclinic, 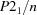

*a* = 11.1058 (4) Å
*b* = 7.2672 (3) Å
*c* = 22.5024 (9) Å
β = 101.0116 (11)°
*V* = 1782.69 (12) Å^3^
*Z* = 4
Mo *K*α radiation
μ = 1.55 mm^−1^
*T* = 223 K
0.20 × 0.19 × 0.08 mm

#### Data collection

Rigaku SCXmini diffractometer
Absorption correction: multi-scan (*REQAB*; Jacobson, 1998 ▶) *T*_min_ = 0.751, *T*_max_ = 0.886
17952 measured reflections
4092 independent reflections
3344 reflections with *F* ^2^ > 2.0σ(*F* ^2^)
*R*_int_ = 0.023

#### Refinement

*R*[*F* ^2^ > 2σ(*F* ^2^)] = 0.027
*wR*(*F* ^2^) = 0.027
*S* = 1.07
3344 reflections
223 parameters
H atoms treated by a mixture of independent and constrained refinement
Δρ_max_ = 0.32 e Å^−3^
Δρ_min_ = −0.36 e Å^−3^

### 

Data collection: *PROCESS-AUTO* (Rigaku, 1998 ▶); cell refinement: *PROCESS-AUTO*; data reduction: *CrystalStructure* (Rigaku/MSC, 2002 ▶); program(s) used to solve structure: *SHELXS97* (Sheldrick, 2008 ▶); program(s) used to refine structure: *CRYSTALS* (Watkin *et al.*, 1999 ▶); molecular graphics: *CrystalStructure*; software used to prepare material for publication: *CrystalStructure*.

## Supplementary Material

Crystal structure: contains datablock(s) General, I. DOI: 10.1107/S1600536812047216/cv5361sup1.cif

Structure factors: contains datablock(s) I. DOI: 10.1107/S1600536812047216/cv5361Isup2.hkl

Additional supplementary materials:  crystallographic information; 3D view; checkCIF report

## Figures and Tables

**Fig. 1.**
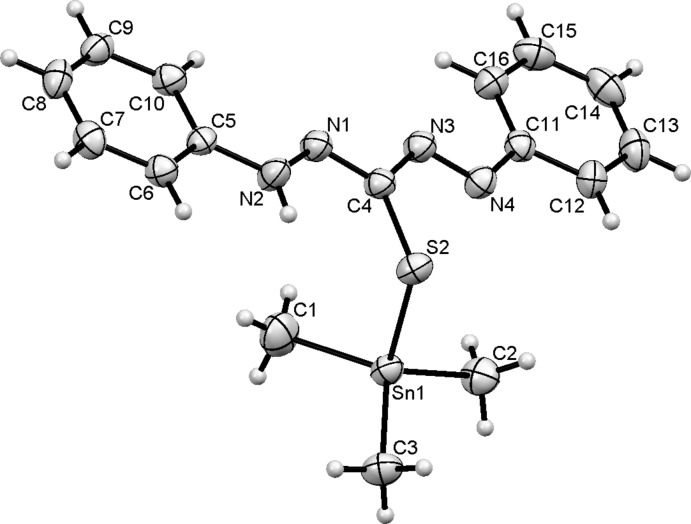
The molecular structure of the title compound showing the atomic numbering and 50% probability displacement ellipsoids.

**Fig. 2.**
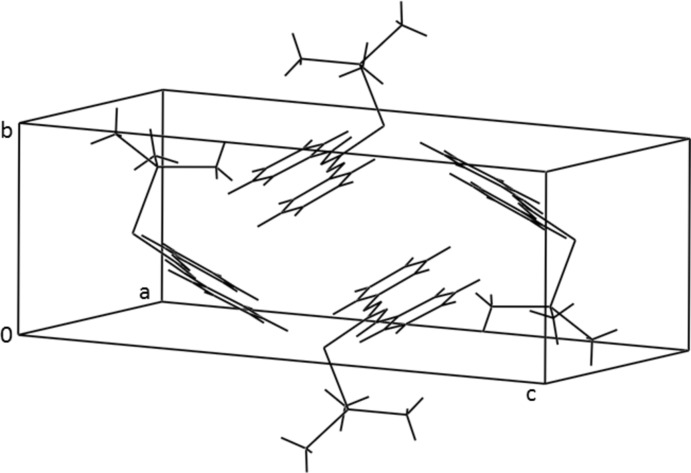
A portion of the molecular packing of the title compound.

## supplementary crystallographic information

### Comment

During a study of the reactions of dimethylamino-trimethyltin, orange crystals of the title compound suitable for X-ray crystallography, were isolated from a diethyl ether solution. The structure revealed distorted tetrahedral coordination geometry around tin, with S—Sn—C bond angles 110.5 (4)°, 105.0 (4)° and 98.0 (9)°. Contrary to most bidentate metal-dithizonate complexes, but with the exception of one ligand in In(HDz)_3_ (Harrowfield *et al.*, 1983) and in an osmium carbonyl cluster compound (Kong & Wong, 1999), coordination of dithizone to trimethyltin(IV) was found to be monodentate, through the sulfur atom alone. The dithizonate ligand clearly illustrates a high degree of planarity, with the ligand backbone being linear, comparable to that of uncoordinated dithizone (Herbstein & Schwotzer, 1984). The Sn—S bond length of 2.4710 (6) Å agrees well with the value of 2.433 (2) Å in a related compound, 4,6-dimethylpyrimidine-2-thione triphenyltin(IV),Ph_3_Sn(Me_2_Pymt), reported by Fernandes *et al.* (2002). The less bulky pyrimidine-thione ligand, however, is bidentately coordinated to Sn through both sulfur and nitrogen, forming a four-membered ring, as opposed to the usual 5-membered metal-dithizonate rings, as seen in PhHgHDz (Von Eschwege *et al.*, 2008). In the case of Me_3_Sn(HDz), the metal lies completely outside the ligand plane, whereas in most other metal dithizonates the carbon-sulfur-metal angle is in the direction of the nitrogen (N4) that does not carry the imine proton, H2 (Laing *et al.*, 1971). The three methyl carbons, being at bond distances of 2.13 (2) Å, hold the metal in the sterically more favourable out-of-plane position. Bond lengths along the ligand backbone are neither typically single nor double bond in character. However, the N3—N4 bond length of 1.267 (9) Å and the N1—C1 bond length of 1.308 (9) Å are close to typical double bond lengths of 1.25 Å and 1.29 Å respectively. Even the N1—N2 bond (1.327 (14) Å), which is expected to be a single bond, has more double bond character than single. N—N single bonds are typically 1.45 Å in length. The N3—C1 bond length of 1.40 (2) Å is shorter than an N—C single bond length of 1.47 Å. Observed deviation from single and double bond distances is further evidence of the high degree of electron delocalization along the dithizonate backbone.

### Experimental

Solvents (AR) purchased from Merck and reagents from Sigma-Aldrich were used without further purification. Dithizone (0.1 g, 0.39 mmol) was dissolved in dry benzene (100 ml) and dimethylamino-trimethyltin (0.082 g, 0.394 mmol) was added under nitrogen. The solvent was removed under reduced pressure, yielding 0.157 g (97%) orange dithizonatotrimethyltin(IV), after crystallization from diethyl ether. The product proved to be unstable in most solvents, except benzene and diethyl ether. M.p. 134°C, λmax/nm (diethyl ether) 442, δH (300 MHz, C_6_D_6_, Spectrum A7)/p.p.m.: 0.47 (6 H, s, 2 × CH_3_), 0.54 (3 H, s, CH_3_), 6.85 – 8.09 (10 H, 3 × m, C_6_H_5_).

### Refinement

The amino H atom was located on a difference map and isotropically refined. C-bound H atoms were placed in calculated positions [C—H = 0.93 Å], and refined as riding, with *U*_iso_(H) = 1.2–1.5*U*_eq_(C).

### Crystal data

**Table:**

[Sn(C_13_H_11_N_4_S)(CH_3_)_3_]	*F*(000) = 840.00
*M**_r_* = 419.11	*D*_x_ = 1.561 Mg m^−^^3^
Monoclinic, *P*2_1_/*n*	Mo *K*α radiation, λ = 0.71075 Å
Hall symbol: -P 2yn	Cell parameters from 16795 reflections
*a* = 11.1058 (4) Å	θ = 3.0–27.6°
*b* = 7.2672 (3) Å	µ = 1.55 mm^−^^1^
*c* = 22.5024 (9) Å	*T* = 223 K
β = 101.0116 (11)°	Needle, orange
*V* = 1782.69 (12) Å^3^	0.20 × 0.19 × 0.08 mm
*Z* = 4	

### Data collection

**Table:**

Rigaku SCXmini diffractometer	3344 reflections with *F*^2^ > 2.0σ(*F*^2^)
Detector resolution: 6.85 pixels mm^-1^	*R*_int_ = 0.023
ω scans	θ_max_ = 27.5°
Absorption correction: multi-scan (*REQAB*; Jacobson, 1998)	*h* = −14→14
*T*_min_ = 0.751, *T*_max_ = 0.886	*k* = −9→9
17952 measured reflections	*l* = −29→28
4092 independent reflections	

### Refinement

**Table:**

Refinement on *F*	H atoms treated by a mixture of independent and constrained refinement
*R*[*F*^2^ > 2σ(*F*^2^)] = 0.027	Chebychev polynomial *w*ith 3 parameters (Carruthers & Watkin, 1979) 3.3785 -1.5365 2.4749
*wR*(*F*^2^) = 0.027	(Δ/σ)_max_ < 0.001
*S* = 1.07	Δρ_max_ = 0.32 e Å^−^^3^
3344 reflections	Δρ_min_ = −0.36 e Å^−^^3^
223 parameters	

### Special details

**Table:**

Geometry. All e.s.d.'s (except the e.s.d. in the dihedral angle between two l.s. planes) are estimated using the full covariance matrix. The cell e.s.d.'s are taken into account individually in the estimation of e.s.d.'s in distances, angles and torsion angles; correlations between e.s.d.'s in cell parameters are only used when they are defined by crystal symmetry. An approximate (isotropic) treatment of cell e.s.d.'s is used for estimating e.s.d.'s involving l.s. planes.
Refinement. Refinement was performed using reflections with *F*^2^ > 3.0 σ(*F*^2^). The weighted *R*-factor(*wR*), goodness of fit (*S*) and *R*-factor (gt) are based on *F*, with *F* set to zero for negative *F*. The threshold expression of *F*^2^ > 2.0 σ(*F*^2^) is used only for calculating *R*-factor (gt).

### Fractional atomic coordinates and isotropic or equivalent isotropic displacement parameters (Å^2^)

**Table:**

	*x*	*y*	*z*	*U*_iso_*/*U*_eq_	
Sn1	0.584750 (10)	0.72516 (2)	0.103690 (10)	0.03181 (4)	
S2	0.48283 (6)	0.42282 (9)	0.08476 (3)	0.03382 (14)	
N1	0.54036 (19)	0.2246 (3)	0.18902 (10)	0.0345 (5)	
N2	0.4294 (2)	0.2702 (3)	0.19760 (10)	0.0376 (5)	
N3	0.69296 (19)	0.2259 (3)	0.13655 (9)	0.0342 (5)	
N4	0.73146 (18)	0.2866 (3)	0.09132 (9)	0.0327 (4)	
C1	0.6418 (3)	0.7378 (5)	0.19937 (13)	0.0556 (9)	
C2	0.7339 (2)	0.7446 (3)	0.05745 (14)	0.0432 (7)	
C3	0.4335 (2)	0.8952 (3)	0.06533 (13)	0.0433 (7)	
C4	0.5753 (2)	0.2867 (3)	0.14066 (11)	0.0313 (5)	
C5	0.3905 (2)	0.2214 (3)	0.25098 (10)	0.0288 (5)	
C6	0.2710 (2)	0.2653 (3)	0.25609 (11)	0.0331 (5)	
C7	0.2291 (2)	0.2216 (3)	0.30840 (12)	0.0371 (6)	
C8	0.3056 (2)	0.1336 (4)	0.35559 (11)	0.0392 (6)	
C9	0.4242 (2)	0.0906 (3)	0.35032 (11)	0.0372 (6)	
C10	0.4679 (2)	0.1334 (3)	0.29838 (11)	0.0342 (6)	
C11	0.8525 (2)	0.2291 (3)	0.08796 (11)	0.0312 (5)	
C12	0.8917 (2)	0.2776 (3)	0.03507 (11)	0.0367 (6)	
C13	1.0091 (2)	0.2330 (4)	0.02757 (13)	0.0456 (7)	
C14	1.0872 (2)	0.1392 (4)	0.07256 (15)	0.0476 (8)	
C15	1.0482 (2)	0.0905 (4)	0.12533 (14)	0.0447 (7)	
C16	0.9316 (2)	0.1346 (3)	0.13339 (12)	0.0372 (6)	
H1	0.6600	0.8589	0.2110	0.067*	
H2	0.5792	0.6946	0.2178	0.067*	
H3	0.7113	0.6652	0.2111	0.067*	
H4	0.7433	0.8663	0.0463	0.052*	
H5	0.8054	0.7050	0.0828	0.052*	
H6	0.7185	0.6712	0.0230	0.052*	
H7	0.3781	0.9004	0.0915	0.052*	
H8	0.4615	1.0131	0.0594	0.052*	
H9	0.3949	0.8462	0.0284	0.052*	
H10	0.2194	0.3239	0.2243	0.040*	
H11	0.1493	0.2516	0.3119	0.044*	
H12	0.2772	0.1034	0.3907	0.047*	
H13	0.4755	0.0319	0.3822	0.045*	
H14	0.5479	0.1037	0.2952	0.041*	
H15	0.8391	0.3401	0.0046	0.044*	
H16	1.0354	0.2663	−0.0078	0.055*	
H17	1.1659	0.1088	0.0674	0.057*	
H18	1.1011	0.0275	0.1556	0.054*	
H19	0.9058	0.1015	0.1689	0.045*	
H20	0.386 (3)	0.335 (4)	0.1727 (14)	0.048 (9)*	

### Atomic displacement parameters (Å^2^)

**Table:**

	*U*^11^	*U*^22^	*U*^33^	*U*^12^	*U*^13^	*U*^23^
Sn1	0.02999 (9)	0.03258 (9)	0.03273 (9)	0.00214 (7)	0.00567 (6)	0.00547 (7)
S2	0.0313 (2)	0.0345 (3)	0.0352 (3)	−0.0008 (2)	0.0050 (2)	0.0076 (2)
N1	0.0357 (10)	0.0341 (10)	0.0367 (10)	0.0043 (8)	0.0142 (8)	0.0069 (8)
N2	0.0350 (10)	0.0453 (12)	0.0352 (10)	0.0091 (9)	0.0133 (8)	0.0130 (9)
N3	0.0337 (9)	0.0347 (10)	0.0360 (10)	0.0022 (8)	0.0112 (8)	0.0070 (8)
N4	0.0312 (9)	0.0357 (10)	0.0319 (9)	−0.0010 (8)	0.0075 (7)	0.0026 (8)
C1	0.0575 (18)	0.067 (2)	0.0384 (14)	0.0051 (15)	−0.0006 (12)	−0.0010 (14)
C2	0.0373 (12)	0.0429 (16)	0.0514 (15)	−0.0042 (10)	0.0133 (11)	0.0021 (11)
C3	0.0409 (14)	0.0390 (14)	0.0482 (15)	0.0051 (11)	0.0044 (12)	0.0079 (11)
C4	0.0313 (11)	0.0298 (10)	0.0343 (11)	0.0013 (9)	0.0102 (9)	0.0046 (9)
C5	0.0316 (10)	0.0256 (10)	0.0308 (10)	−0.0018 (9)	0.0096 (8)	0.0020 (8)
C6	0.0315 (11)	0.0331 (12)	0.0348 (11)	0.0006 (9)	0.0068 (9)	0.0012 (9)
C7	0.0305 (11)	0.0404 (12)	0.0432 (12)	−0.0035 (10)	0.0144 (9)	−0.0032 (11)
C8	0.0439 (14)	0.0459 (15)	0.0302 (12)	−0.0090 (11)	0.0134 (10)	−0.0007 (10)
C9	0.0442 (14)	0.0370 (13)	0.0284 (11)	−0.0020 (10)	0.0020 (10)	0.0046 (9)
C10	0.0301 (11)	0.0350 (12)	0.0376 (12)	0.0017 (9)	0.0070 (9)	0.0033 (10)
C11	0.0339 (11)	0.0290 (11)	0.0313 (10)	−0.0021 (9)	0.0077 (8)	−0.0034 (9)
C12	0.0381 (12)	0.0432 (13)	0.0306 (11)	−0.0008 (11)	0.0108 (9)	−0.0017 (10)
C13	0.0467 (14)	0.0540 (17)	0.0416 (13)	−0.0017 (13)	0.0221 (11)	−0.0056 (12)
C14	0.0363 (14)	0.0462 (16)	0.0628 (18)	0.0056 (11)	0.0159 (12)	−0.0108 (14)
C15	0.0404 (14)	0.0387 (14)	0.0528 (16)	0.0080 (11)	0.0036 (12)	0.0023 (12)
C16	0.0411 (13)	0.0363 (13)	0.0346 (12)	0.0017 (10)	0.0080 (10)	0.0049 (10)

### Geometric parameters (Å, º)

**Table:**

Sn1—S2	2.4710 (6)	C15—C16	1.380 (4)
Sn1—C1	2.127 (2)	N2—H20	0.82 (3)
Sn1—C2	2.123 (3)	C1—H1	0.930
Sn1—C3	2.130 (2)	C1—H2	0.930
S2—C4	1.766 (2)	C1—H3	0.930
N1—N2	1.325 (3)	C2—H4	0.930
N1—C4	1.304 (3)	C2—H5	0.930
N2—C5	1.398 (3)	C2—H6	0.930
N3—N4	1.257 (3)	C3—H7	0.930
N3—C4	1.400 (3)	C3—H8	0.930
N4—C11	1.423 (3)	C3—H9	0.930
C5—C6	1.391 (3)	C6—H10	0.930
C5—C10	1.391 (3)	C7—H11	0.930
C6—C7	1.383 (3)	C8—H12	0.930
C7—C8	1.383 (3)	C9—H13	0.930
C8—C9	1.381 (4)	C10—H14	0.930
C9—C10	1.384 (3)	C12—H15	0.930
C11—C12	1.389 (3)	C13—H16	0.930
C11—C16	1.395 (3)	C14—H17	0.930
C12—C13	1.385 (4)	C15—H18	0.930
C13—C14	1.380 (4)	C16—H19	0.930
C14—C15	1.385 (4)		
			
C3···C4^i^	3.526 (3)	H7···C8^iv^	3.204
C4···C3^ii^	3.526 (3)	H7···H4^viii^	3.565
C13···C13^iii^	3.599 (4)	H7···H11^iv^	2.499
C13···C14^iii^	3.552 (4)	H7···H12^iv^	2.839
C14···C13^iii^	3.552 (4)	H7···H15^v^	3.399
Sn1···H11^iv^	3.506	H7···H17^x^	2.766
S2···H6^v^	3.045	H8···S2^i^	3.033
S2···H8^ii^	3.033	H8···N1^i^	3.266
S2···H12^iv^	3.314	H8···N3^i^	3.211
N1···H1^ii^	2.970	H8···N4^i^	3.554
N1···H3^vi^	3.237	H8···C2^viii^	3.542
N1···H7^ii^	3.479	H8···C3^viii^	3.306
N1···H8^ii^	3.266	H8···C4^i^	2.831
N2···H7^ii^	3.568	H8···H4^viii^	3.088
N3···H1^ii^	3.208	H8···H6^viii^	3.359
N3···H3^vi^	3.419	H8···H8^viii^	2.963
N3···H4^ii^	3.420	H8···H9^viii^	2.948
N3···H8^ii^	3.211	H8···H17^x^	3.395
N4···H4^ii^	3.228	H9···N4^v^	2.952
N4···H8^ii^	3.554	H9···C11^v^	3.458
N4···H9^v^	2.952	H9···C12^v^	3.354
C1···H11^iv^	3.196	H9···H4^viii^	2.926
C1···H14^vii^	3.561	H9···H8^viii^	2.948
C1···H19^vii^	3.263	H9···H12^iv^	3.379
C2···H8^viii^	3.542	H9···H15^v^	2.898
C2···H16^ix^	2.985	H9···H17^x^	3.426
C2···H17^ix^	3.386	H10···C5^iv^	3.225
C3···H4^viii^	3.360	H10···C6^iv^	3.236
C3···H8^viii^	3.306	H10···C7^iv^	3.063
C3···H11^iv^	3.247	H10···C8^iv^	2.861
C3···H12^iv^	3.441	H10···C9^iv^	2.845
C3···H15^v^	3.570	H10···C10^iv^	3.039
C3···H17^x^	3.362	H10···C15^xii^	3.136
C4···H1^ii^	3.534	H10···H11^iv^	3.592
C4···H8^ii^	2.831	H10···H12^iv^	3.296
C5···H10^xi^	3.225	H10···H13^iv^	3.276
C5···H18^vii^	3.050	H10···H14^iv^	3.555
C6···H10^xi^	3.236	H10···H18^xii^	2.826
C6···H18^xii^	3.168	H10···H18^vii^	3.378
C6···H18^vii^	2.919	H11···Sn1^xi^	3.506
C7···H2^xi^	3.367	H11···C1^xi^	3.196
C7···H7^xi^	3.034	H11···C3^xi^	3.247
C7···H10^xi^	3.063	H11···H1^xi^	3.464
C7···H18^vii^	2.927	H11···H2^xi^	2.532
C7···H20^xi^	3.15 (3)	H11···H7^xi^	2.499
C8···H7^xi^	3.204	H11···H10^xi^	3.592
C8···H10^xi^	2.861	H11···H18^vii^	3.386
C8···H15^xiii^	3.308	H11···H20^xi^	3.079
C8···H18^vii^	3.071	H12···S2^xi^	3.314
C8···H20^xi^	3.02 (3)	H12···C3^xi^	3.441
C9···H5^vi^	3.202	H12···C12^xiii^	3.368
C9···H10^xi^	2.845	H12···H7^xi^	2.839
C9···H16^xiii^	3.361	H12···H9^xi^	3.379
C9···H18^vii^	3.188	H12···H10^xi^	3.296
C10···H5^vi^	3.345	H12···H15^xiii^	2.558
C10···H10^xi^	3.039	H12···H16^xiii^	3.440
C10···H18^vii^	3.189	H12···H18^vii^	3.598
C11···H4^ii^	2.978	H12···H20^xi^	2.853
C11···H9^v^	3.458	H13···C11^vi^	2.907
C11···H13^vii^	2.907	H13···C12^vi^	2.830
C12···H4^ii^	3.446	H13···C13^vi^	2.956
C12···H9^v^	3.354	H13···C14^vi^	3.152
C12···H12^xiv^	3.368	H13···C15^vi^	3.221
C12···H13^vii^	2.830	H13···C16^vi^	3.109
C12···H16^ix^	3.494	H13···H5^vi^	2.719
C13···H4^ix^	3.548	H13···H10^xi^	3.276
C13···H5^ix^	3.545	H13···H15^vi^	3.266
C13···H6^ix^	3.499	H13···H15^xiii^	3.514
C13···H13^vii^	2.956	H13···H16^vi^	3.444
C13···H17^iii^	3.596	H13···H16^xiii^	2.845
C14···H4^ix^	3.553	H14···C1^vi^	3.561
C14···H6^ix^	3.599	H14···H1^ii^	3.033
C14···H13^vii^	3.152	H14···H2^ii^	3.496
C14···H16^iii^	3.450	H14···H3^vi^	2.742
C15···H10^xv^	3.136	H14···H5^vi^	3.004
C15···H13^vii^	3.221	H14···H10^xi^	3.555
C16···H2^vi^	3.401	H15···C3^v^	3.570
C16···H4^ii^	3.232	H15···C8^xiv^	3.308
C16···H5^ii^	3.521	H15···H7^v^	3.399
C16···H13^vii^	3.109	H15···H9^v^	2.898
H1···N1^i^	2.970	H15···H12^xiv^	2.558
H1···N3^i^	3.208	H15···H13^vii^	3.266
H1···C4^i^	3.534	H15···H13^xiv^	3.514
H1···H3^vii^	3.019	H15···H16^ix^	3.177
H1···H11^iv^	3.464	H16···C2^ix^	2.985
H1···H14^i^	3.033	H16···C9^xiv^	3.361
H1···H19^i^	3.529	H16···C12^ix^	3.494
H1···H19^vii^	3.477	H16···C14^iii^	3.450
H2···C7^iv^	3.367	H16···H4^ix^	2.921
H2···C16^vii^	3.401	H16···H5^ix^	2.676
H2···H11^iv^	2.532	H16···H6^ix^	2.855
H2···H14^i^	3.496	H16···H12^xiv^	3.440
H2···H19^vii^	2.612	H16···H13^vii^	3.444
H3···N1^vii^	3.237	H16···H13^xiv^	2.845
H3···N3^vii^	3.419	H16···H15^ix^	3.177
H3···H1^vi^	3.019	H16···H16^ix^	3.519
H3···H14^vii^	2.742	H17···C2^ix^	3.386
H3···H19^vii^	3.247	H17···C3^xvi^	3.362
H4···N3^i^	3.420	H17···C13^iii^	3.596
H4···N4^i^	3.228	H17···H4^ix^	2.931
H4···C3^viii^	3.360	H17···H6^ix^	3.057
H4···C11^i^	2.978	H17···H7^xvi^	2.766
H4···C12^i^	3.446	H17···H8^xvi^	3.395
H4···C13^ix^	3.548	H17···H9^xvi^	3.426
H4···C14^ix^	3.553	H17···H20^xv^	3.479
H4···C16^i^	3.232	H18···C5^vi^	3.050
H4···H7^viii^	3.565	H18···C6^xv^	3.168
H4···H8^viii^	3.088	H18···C6^vi^	2.919
H4···H9^viii^	2.926	H18···C7^vi^	2.927
H4···H16^ix^	2.921	H18···C8^vi^	3.071
H4···H17^ix^	2.931	H18···C9^vi^	3.188
H4···H19^i^	3.448	H18···C10^vi^	3.189
H5···C9^vii^	3.202	H18···H10^xv^	2.826
H5···C10^vii^	3.345	H18···H10^vi^	3.378
H5···C13^ix^	3.545	H18···H11^vi^	3.386
H5···C16^i^	3.521	H18···H12^vi^	3.598
H5···H13^vii^	2.719	H19···C1^vi^	3.263
H5···H14^vii^	3.004	H19···H1^ii^	3.529
H5···H16^ix^	2.676	H19···H1^vi^	3.477
H5···H19^i^	3.532	H19···H2^vi^	2.612
H6···S2^v^	3.045	H19···H3^vi^	3.247
H6···C13^ix^	3.499	H19···H4^ii^	3.448
H6···C14^ix^	3.599	H19···H5^ii^	3.532
H6···H8^viii^	3.359	H20···C7^iv^	3.15 (3)
H6···H16^ix^	2.855	H20···C8^iv^	3.02 (3)
H6···H17^ix^	3.057	H20···H11^iv^	3.079
H7···N1^i^	3.479	H20···H12^iv^	2.853
H7···N2^i^	3.568	H20···H17^xii^	3.479
H7···C7^iv^	3.034		
			
S2—Sn1—C1	104.53 (9)	H1—C1—H2	109.5
S2—Sn1—C2	110.46 (7)	H1—C1—H3	109.5
S2—Sn1—C3	98.40 (7)	H2—C1—H3	109.5
C1—Sn1—C2	112.56 (12)	Sn1—C2—H4	109.5
C1—Sn1—C3	116.40 (12)	Sn1—C2—H5	109.5
C2—Sn1—C3	113.04 (11)	Sn1—C2—H6	109.5
Sn1—S2—C4	100.97 (8)	H4—C2—H5	109.5
N2—N1—C4	117.9 (2)	H4—C2—H6	109.5
N1—N2—C5	120.7 (2)	H5—C2—H6	109.5
N4—N3—C4	114.15 (19)	Sn1—C3—H7	109.5
N3—N4—C11	114.26 (19)	Sn1—C3—H8	109.5
S2—C4—N1	124.29 (18)	Sn1—C3—H9	109.5
S2—C4—N3	123.56 (18)	H7—C3—H8	109.5
N1—C4—N3	112.1 (2)	H7—C3—H9	109.5
N2—C5—C6	118.0 (2)	H8—C3—H9	109.5
N2—C5—C10	121.9 (2)	C5—C6—H10	120.0
C6—C5—C10	120.1 (2)	C7—C6—H10	120.0
C5—C6—C7	120.0 (2)	C6—C7—H11	119.9
C6—C7—C8	120.2 (2)	C8—C7—H11	119.9
C7—C8—C9	119.6 (2)	C7—C8—H12	120.2
C8—C9—C10	121.1 (2)	C9—C8—H12	120.2
C5—C10—C9	119.1 (2)	C8—C9—H13	119.5
N4—C11—C12	115.2 (2)	C10—C9—H13	119.5
N4—C11—C16	125.1 (2)	C5—C10—H14	120.5
C12—C11—C16	119.7 (2)	C9—C10—H14	120.5
C11—C12—C13	120.1 (2)	C11—C12—H15	119.9
C12—C13—C14	120.0 (2)	C13—C12—H15	119.9
C13—C14—C15	120.0 (2)	C12—C13—H16	120.0
C14—C15—C16	120.5 (2)	C14—C13—H16	120.0
C11—C16—C15	119.6 (2)	C13—C14—H17	120.0
N1—N2—H20	119 (2)	C15—C14—H17	120.0
C5—N2—H20	120 (2)	C14—C15—H18	119.7
Sn1—C1—H1	109.5	C16—C15—H18	119.7
Sn1—C1—H2	109.5	C11—C16—H19	120.2
Sn1—C1—H3	109.5	C15—C16—H19	120.2
			
C(1)—Sn(1)—S(2)—C(4)	−36.42 (13)	N(2)—C(5)—C(10)—C(9)	179.3 (2)
C(2)—Sn(1)—S(2)—C(4)	84.89 (12)	C(6)—C(5)—C(10)—C(9)	−0.0 (3)
C(3)—Sn(1)—S(2)—C(4)	−156.59 (12)	C(10)—C(5)—C(6)—C(7)	0.1 (2)
Sn(1)—S(2)—C(4)—N(1)	108.4 (2)	C(5)—C(6)—C(7)—C(8)	−0.3 (3)
Sn(1)—S(2)—C(4)—N(3)	−74.7 (2)	C(6)—C(7)—C(8)—C(9)	0.4 (4)
N(2)—N(1)—C(4)—S(2)	−1.7 (3)	C(7)—C(8)—C(9)—C(10)	−0.3 (4)
N(2)—N(1)—C(4)—N(3)	−178.9 (2)	C(8)—C(9)—C(10)—C(5)	0.1 (3)
C(4)—N(1)—N(2)—C(5)	−174.5 (2)	N(4)—C(11)—C(12)—C(13)	178.3 (2)
N(1)—N(2)—C(5)—C(6)	−176.9 (2)	N(4)—C(11)—C(16)—C(15)	−178.3 (2)
N(1)—N(2)—C(5)—C(10)	3.8 (3)	C(12)—C(11)—C(16)—C(15)	0.1 (3)
N(4)—N(3)—C(4)—S(2)	4.5 (3)	C(16)—C(11)—C(12)—C(13)	−0.3 (3)
N(4)—N(3)—C(4)—N(1)	−178.3 (2)	C(11)—C(12)—C(13)—C(14)	0.4 (4)
C(4)—N(3)—N(4)—C(11)	178.29 (19)	C(12)—C(13)—C(14)—C(15)	−0.3 (4)
N(3)—N(4)—C(11)—C(12)	173.2 (2)	C(13)—C(14)—C(15)—C(16)	0.1 (3)
N(3)—N(4)—C(11)—C(16)	−8.3 (3)	C(14)—C(15)—C(16)—C(11)	−0.0 (3)
N(2)—C(5)—C(6)—C(7)	−179.2 (2)		

Symmetry codes: (i) *x*, *y*+1, *z*; (ii) *x*, *y*−1, *z*; (iii) −*x*+2, −*y*, −*z*; (iv) −*x*+1/2, *y*+1/2, −*z*+1/2; (v) −*x*+1, −*y*+1, −*z*; (vi) −*x*+3/2, *y*−1/2, −*z*+1/2; (vii) −*x*+3/2, *y*+1/2, −*z*+1/2; (viii) −*x*+1, −*y*+2, −*z*; (ix) −*x*+2, −*y*+1, −*z*; (x) *x*−1, *y*+1, *z*; (xi) −*x*+1/2, *y*−1/2, −*z*+1/2; (xii) *x*−1, *y*, *z*; (xiii) *x*−1/2, −*y*+1/2, *z*+1/2; (xiv) *x*+1/2, −*y*+1/2, *z*−1/2; (xv) *x*+1, *y*, *z*; (xvi) *x*+1, *y*−1, *z*.
